# Treatment of Angular Deformity and Limb Length Discrepancy With a Retrograde Femur Magnetic Intramedullary Nail: A Fixator-assisted, Blocking Screw Technique

**DOI:** 10.5435/JAAOSGlobal-D-23-00053

**Published:** 2023-05-18

**Authors:** Erik J. Geiger, Adam D. Geffner, S. Robert Rozbruch, Austin T. Fragomen

**Affiliations:** From the Department of Orthopedic Surgery, The Rothman Institute, Thomas Jefferson University, Philadelphia, PA (Dr. Geiger) and the Department of Limb Lengthening and Complex Reconstruction, Hospital for Special Surgery, New York, NY (Mr. Geffner, Dr. Rozbruch, Dr. Fragomen).

## Abstract

**Background:**

Fixator-assisted nailing techniques that incorporate magnetic internal lengthening nails (MILNs) permit acute deformity correction and then gradual limb lengthening without needing postoperative external fixators.

**Purposes:**

We sought to investigate the safety and accuracy of a fixator-assisted, blocking screw technique using retrograde MILNs for the correction of LLD and limb malalignment.

**Methods:**

Forty-one patients (13 patients with genu varum and 28 patients with genu valgum) with LLD treated with fixator-assisted, blocking screw retrograde MILN reconstruction were included. Preoperative LLD, mechanical axis deviation, and joint orientation angles were compared with values at the end of treatment, and bone healing indices were calculated. Perioperative complications were tracked.

**Results:**

Preoperatively, the mean mechanical lateral distal femoral angle of the varus cohort was 98 ± 12°, whereas the mean lateral distal femoral angle of the valgus cohort was 82±4°. Both cohorts had an average 3-cm LLD. 99% of the planned limb lengthening was achieved. Final LDFAs were 91 ± 6° and 89 ± 4° in the varus and valgus cohorts, respectively, and the limb mechanical axis angles were normalized. 10 patients underwent a total of 21 returns to the operating room. Most commonly, this involved percutaneous injection of bone marrow aspirate concentrate to bone regenerate exhibiting delayed union (6 patients).

**Conclusions:**

The use of a retrograde MILN with a fixator-assisted, blocking screw technique is an effective means of acute deformity correction and gradual limb lengthening through minimal incisions. The accuracy of deformity correction relies on intraoperative execution of the appropriate nail start site, osteotomy location, and placement of blocking screws.

Patients with concomitant leg length discrepancy (LLD) and limb malalignment have been treated with external fixation devices based on the principles of the Ilizarov method and distraction osteogenesis.^1,2,3,4^ However, prolonged periods in external fixators are associated with complications, including pain, pin site infections requiring antibiotics, joint contractures, and refracture after frame removal.^5^ Multiple techniques have been developed to obtain corrections while limiting time spent in external frames—such as lengthening over a nail (LON)^6^ or lengthening and then nailing.^7^ Then, the development of the Precice intramedullary limb lengthening system (NuVasive Orthopaedics Inc) revolutionized the treatment of length discrepancy and deformity about the knee.^8^ This magnetic internal lengthening nail (MILN) demonstrated excellent reliability, safety, and accuracy—superior to that of its predecessors^9^—and largely replaced LON and lengthening and then nailing.^10^

Although MILNs can address LLD, these intramedullary devices are not able to additionally correct limb deformities. Fortunately, fixator-assisted nailing (FAN) and plating (FAP) techniques were separately developed to help surgeons accurately correct deformities in the operating room through osteotomies.^11,12^ Although an external fixator is applied in the operating room to hold acute deformity correction, it is removed after the correction is stabilized with internal implants. Combining FAN with MILNs allows surgeons to conduct acute deformity correction and then gradual limb lengthening and to treat complex deformities without postoperative external fixation. While FAN can be conducted using an antegrade or retrograde approach, indications for a retrograde approach include (1) a distal femoral deformity with planned osteotomy distal to the femoral isthmus, (2) proximal femoral implants or prosthesis blocking an antegrade approach, or (3) the preference to avoid exacerbating underlying hip abductor weakness.^13^ The challenges of a retrograde approach lie in the width of the distal femur; hence, the success of this approach hinges on executing the proper nail start site and trajectory in the distal fragment plus the accurate placement of blocking screws to both correct deformity and prevent the introduction of new deformity during lengthening. The purpose of this study was to investigate the safety and accuracy of the fixator-assisted, blocking screw technique using a retrograde MILN for the correction of LLD and coronal plane limb malalignment.

## Methods

### Study Cohort

After obtaining institutional review board approval, our patient registry was used to identify the study population retrospectively. Patients were included if they had undergone coronal plane deformity and LLD correction using a retrograde Precice MILN with a fixator-assisted, blocking screw technique. Patients were excluded if they were treated for multiplanar deformity or if treatment with a retrograde MILN was part of a multistage reconstruction of the ipsilateral femur and/or tibia. Patient demographics including age at surgery, sex, diagnosis, and surgical interventions were obtained from the electronic medical record (EMR). Additional information including implant sizes, the rate and rhythm of transport, and bone healing metrics (defined below) were obtained from the EMR or measured on calibrated radiographs.

### Outcomes and Definitions

The distraction time (days) was calculated from the initiation of nail distraction until the planned lengthening was achieved. The bone healing time (days) was calculated from the initiation of nail distraction until consolidation of the regenerate bone. Consolidation of the regenerate was defined radiographically as at least 2 mm of continuous bone bridging three of four cortices. The Bone Healing Index (BHI) days/cm equaled the bone healing time divided by the distraction gap length. Preoperative radiographic measurements were compared with the corresponding postoperative values and included the mechanical lateral distal femoral angle (LDFA), medial proximal tibial angle, tibiofemoral or mechanical axis angle (MAA), mechanical axis deviation (MAD), and LLD. The total lengthening achieved was compared with the preoperative plan. Difficulties encountered during treatment were subclassified as problems, obstacles, or sequelae according to the criteria by Paley.^14^ Problems represented difficulties that required no surgical intervention to resolve, whereas obstacles represented difficulties that required a surgical intervention. All intraoperative injuries and all problems during limb lengthening that were not resolved before the end of treatment were considered sequelae.

### Statistical Analysis

Descriptive statistics were used to summarize data. Two-sample Student *t*-tests (assuming unequal variances) were used to compare means between the varus and valgus cohorts, and paired two-sample Student *t*-tests were used to compare preoperative and postoperative means in each cohort. These tests were conducted using Microsoft Excel 2019 (Redmond, WA). Statistical significance was defined as *P* < 0.05.

### Surgical Technique

**Figure 1:**
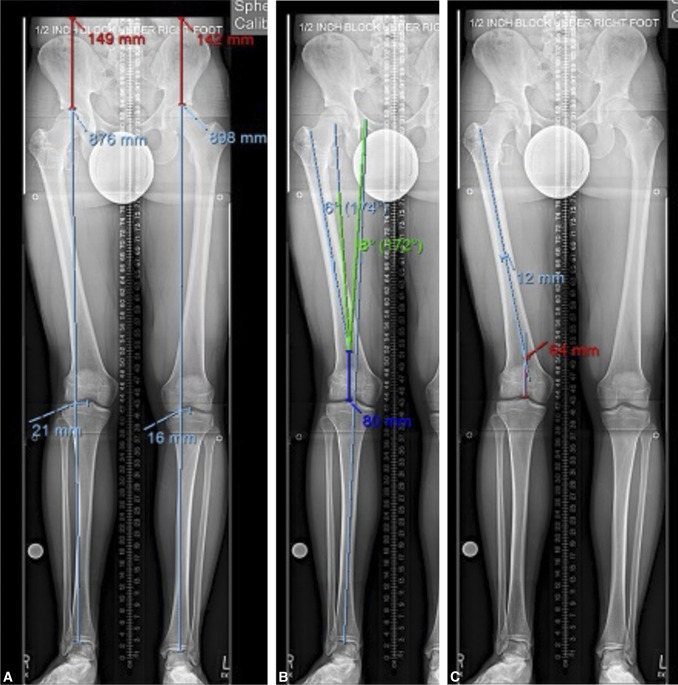
Long-standing radiographs showing preoperative 20-mm LLD plus 21-mm lateral mechanical axis deviation (**A**) with 8° mechanical axis valgus deformity (**B**). Mechanical axis planning is then transferred to anatomic axis planning knowing the nail is restricted to the intramedullary space proximally (**C**).

**Figure 2:**
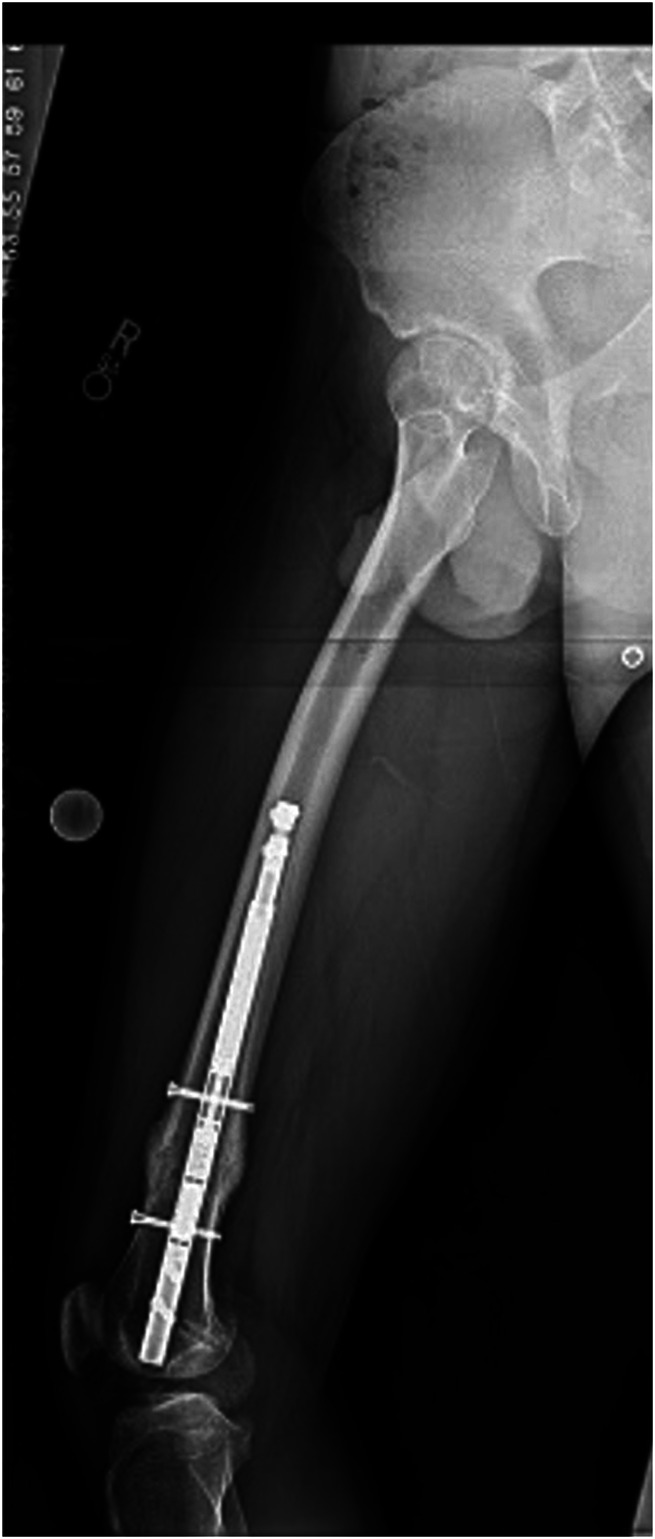
Radiograph showing that selecting the appropriate nail length is vital such that it will end distal to the anterior femoral bow and avoid anterior cortical impingement during insertion of the straight magnetic internal lengthening nails.

Our preferred method for reconstruction using a retrograde MILN has been detailed previously.^13^ In brief, achieving a successful outcome begins with a detailed preoperative plan. Calibrated 51-inch standing hip-to-ankle radiographs are obtained; lower extremity lengths, segment lengths (tibia and femur), joint orientation angles, and mechanical axis deviations are measured bilaterally; and the center of rotation of angulation is identified according to methods described by Paley and Tetsworth^15^ (Figure 1, A and B). The osteotomy level is selected ensuring that enough bone stock is available in the distal segment for MILN fixation. The mechanical axis deformity analysis is then translated to anatomic axis surgical planning because the MILN is restricted to an intramedullary position in the proximal fragment (Figure 1C). This is illustrated in detail in the technique publication by Fragomen et al^13^ The minimum length of the MILN is preoperatively determined by a simple calculation equal to the distance in millimeters from the nail insertion site to the osteotomy level plus the planned lengthening distance plus 50 mm plus 30 mm. The final numbers come from our desire to have 50 mm of the thick housing of the MILN in the proximal fragment at the end of lengthening plus the 30-mm thin tip of these nails; this assures adequate control of the proximal fragment and minimizes risk of mechanical failure of the telescopic junction of the nail, should it end within the regenerate at the conclusion of lengthening. Calibrated 36-inch lateral femur radiographs are also evaluated, and the nail length is selected such that it will end distal to the anterior femoral bow, thus avoiding anterior cortical impingement by the straight MILN (Figure 2). The largest nail diameter that can be accommodated by reasonable reaming of the distal diaphysis is selected based on the surgeon's judgement.

For our fixator-assisted, blocking screw technique, special attention is paid to the nail start site and its direction in the distal fragment at this stage of the plan. The start site may be slightly lateral of the notch center in valgus knees and slightly medial of center in varus knees. Of equal importance is the templated location of blocking screws because it is the nail start plus the blocking screw location that will ultimately determine deformity correction. The locations of blocking screws are guided by the “reverse rule of thumbs”^16^ for angular correction, and their exact location in the bone is based on the nail diameter and cortical width. 5.0-mm blocking screws are placed lateral of the nail trajectory when correcting valgus and are placed medial of the nail trajectory when correcting varus. Screws are placed 1-2 cm away from the planned osteotomy to avoid propagation of the osteotomy into a screw path compromising its purchase. In addition to correcting deformity, blocking screws are also placed to resist lengthening-induced deformity, particularly flexion/procurvatum. If the nail is not abutting the posterior cortex at the conclusion of the surgery, a posterior-blocking screw will be placed that will resist flexion.

**Figure 3:**
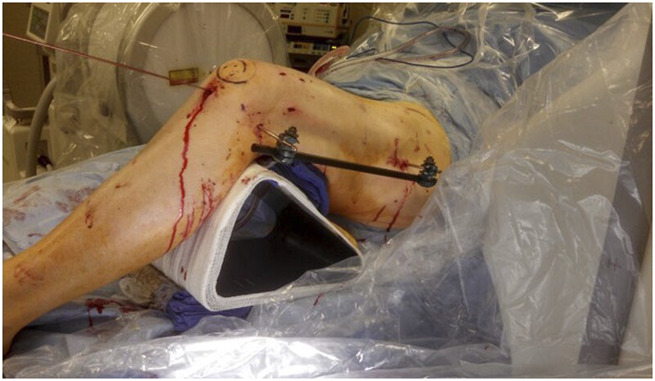
Photograph showing that the acute deformity correction is temporarily held with an external fixator connected to the Schanz pins placed at the start of the procedure to mark femoral rotation.

**Figure 4:**
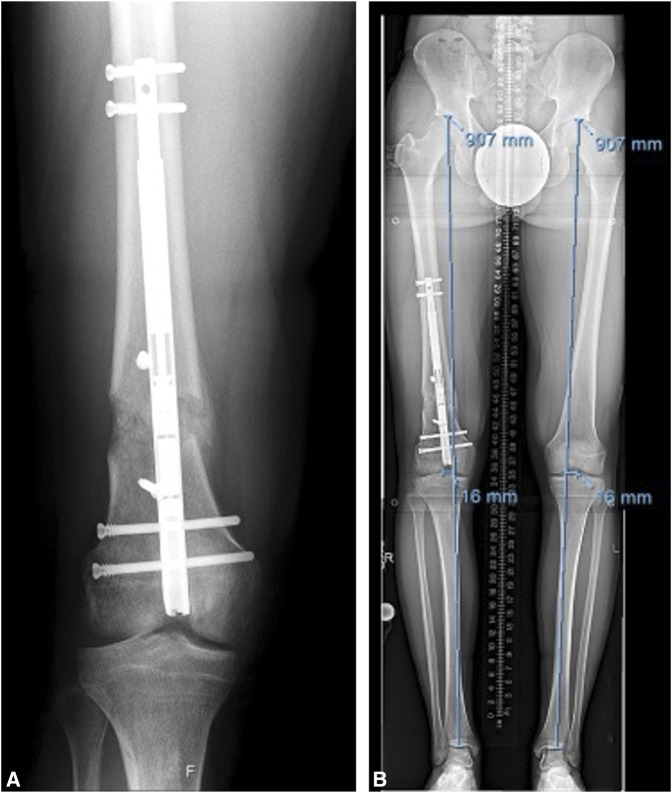
Femural radiographs showing the blocking screws critical to this valgus correction and consolidation of the regenerate (**A**). Hip-to-ankle radiograph showing equalization of the patient's leg lengths and correction of the mechanical axis deviation, symmetric to his other side, after treatment (**B**).

6-mm Schanz pins are placed from lateral out of the path of the nail to mark femoral rotation. Once the nail start site has been opened and the blocking screws have been placed, the multiple drill-hole osteotomy is completed with an osteotome. A full but provisional angular deformity correction is conducted manually, and the reduction is temporarily held with an external fixator connected to the Schanz pins (Figure 3). Flexible reaming proceeds, and the templated nail is inserted in the usual way. Before fully seating the nail, the fixator is loosened so that the MILN trajectory plus blocking screws work in concert to achieve the ultimate deformity correction consistent with the preoperative plan. Before guided distal and proximal freehand interlocking of the nail, the limb alignment can be evaluated with a radiopaque alignment rod, ruler, or electrocautery cord. Any residual alignment adjustments can be made by manipulating the external fixator if needed. Careful attention is simultaneously paid to maintaining the femoral version as originally marked. Once the deformity correction has been stabilized by static interlocking of the MILN, the external fixator is removed, the incisions are washed and closed, and the location of the MILN magnet is marked on the skin. The Precice nail creates distraction through a series of gears that drive an inner telescopic rod. These gears turn in response to an external magnetic field applied by the manufacturer's external remote control device.^10^ Our distraction protocol has been 0.2 mm four times daily beginning on postoperative day 7 in this cohort. Postoperative weight-bearing is determined by the MILN diameter. Vitamin D levels are checked, and all patients are supplemented with daily vitamin D (5,000 IU) and calcium (1,250 mg) postoperatively except in rare instances where hypervitaminosis was discovered preoperatively. Patient factors representing contraindications to this technique are active infection and current smoking, both of which require treatment before this elective reconstruction. A representative result early after consolidation is shown in Figures 4, A and B.

## Results

### Patient Characteristics

Thirteen patients having distal femoral varus and 28 patients with distal femoral valgus were included (Table 1). The mean ages were 35 ± 12 years and 26 ± 10 years (*P* = 0.03), respectively. As expected, the varus and valgus cohort differed markedly in measurements of their joint orientation angles and MAD. The varus cohort had a mean LDFA of 98 ± 12° and a MAD of 31 ± 22 mm medial, whereas the valgus cohort had a mean LDFA of 82 ± 4° and a MAD of 19 ± 9 mm lateral. The average planned lengthening was 43 ± 22 mm for the varus group and 29 ± 12 mm for the valgus group (*P* = 0.05).

**Table 1:** Demographics and Characteristics of Patients Treated for Varus or Valgus Deformity and Leg Length Discrepancy Using an Internal Retrograde Femoral Magnetic Lengthening Nail

	Varus Cohort	Valgus Cohort	
	n = 13	n = 28	*P* value^a^
Sex (n, %)			
Men	8 (73%)	14 (52%)	
Women	3 (27%)	13 (48%)	
Age (years) Mean ± SD	34.9 ± 11.7	25.5 ± 10.2	**0.03**
Follow-up (months) Mean ± SD	26.3 ± 17.3	25.2 ± 15.3	0.86
Etiology			
Congenital	5	14	
Trauma	3	10	
Others	2	3	
Unknown	1	1	
Direct LLD (mm) Mean ± SD	27.3 ± 18.8	25.2 ± 11.1	0.74
Indirect LLD (mm) Mean ± SD	29.1 ± 15.6	26.3 ± 12.1	0.60
MAD (mm)	Medial (+)	Lateral (−)	**<0.001**
Mean ± SD	31.2 ± 21.5	−19.0 ± 8.7	
MAD > 5 mm (n, %)^b^	13 (100%)	28 (100%)	
Mechanical axis angle	Deg. Varus (+)	Deg. Valgus (−)	**<0.001**
Mean ± SD	10.34 ± 9.0	−6.2 ± 2.3	
LDFA (degrees) Mean ± SD	98.4 ± 11.8	82.5 ± 3.9	**<0.001**
MPTA (degrees) Mean ± SD	88.2 ± 5.2	88.6 ± 2.8	0.76
Planned lengthening (mm) Mean ± SD	43.2 ± 21.8	29 ± 11.7	**0.05**
Latency (days) Mean ± SD	6.6 ± 1.0	6.3 ± 1.3	0.37
Rate (mm/day) Mean ± SD	0.8 ± 0.1	0.8 ± 0.1	0.67
Rhythm (events per day) Mean ± SD	3.2 ± 0.5	3.4 ± 0.6	0.41
Blocking screws (n) Mean ± SD	1.8 ± 0.4	1.8 ± 0.7	0.92

LLD = leg length discrepancy, LDFA = lateral distal femoral angle, MPTA = medial proximal tibial angle, MAD = mechanical axis deviation

a The two-sample Student *t*-test assuming unequal variances (two-tailed *P* values).

b All MAD for varus knees were medial to the knee midline; all MAD for valgus knees were lateral to the midline.

### Radiographic Outcomes

Excellent precision and control over lengthening was demonstrated because on average 99% and 100% of the planned lengthening in the varus and valgus cohort, respectively, was achieved. The BHIs were not statistically different, measuring 28 ± 6 days/cm and 35 ± 22 days/cm (Table 2). Normalization of the LDFA (normal range 88-90°)^17^ and MAA (normal ∼2° varus)^18^ were achieved in both cohorts. Similarly, given a normal MAD range of up to 10 mm from midline,^17^ MAD were adequately corrected in each group (Table 3). Although these final parameters were not different from each other from an intergroup comparison (as expected), the changes in LDFA, MAA, and MAD were markedly changed from their preoperative means (Supplemental Table 1, http://links.lww.com/JG9/A283). In the nine varus patients with residual MAD >5 mm after treatment, the average preoperative MAD was 32 mm with a maximum deviation of up to 69 mm medially. In the 12 valgus patients with residual MAD >5 mm after treatment, the average preoperative MAD was 22 mm and went up to 35 mm laterally.

**Table 2:** Total Lengthening Achieved and the Average Bone Healing Index for Each Study Cohort After Treatment of Coronal Malalignment and Leg Length Discrepancy

	Varus Cohort	Valgus Cohort	
	n = 13	n = 28	*P* value
Total lengthening (mm) Mean ± SD	43 ± 22	29 ± 11	0.06
BHI (days/cm) Mean ± SD	28 ± 6	35 + 22	0.16

BHI = Bone Healing Index

**Table 3:** Clinical and Radiographic Outcomes After Using an Internal Retrograde Femoral Magnetic Lengthening Nail for Coronal Deformity and Leg Length Discrepancy Correction

	Varus Cohort	Valgus Cohort	
	n = 13	n = 28	*P* value
Total lengthening (mm) Mean ± SD	42.6 ± 22.1	28.8 ± 11.3	0.06
BHI (days/cm) Mean ± SD	28.2 ± 6.1	34.8 + 22.2	0.16
Final direct LLD (mm) Mean ± SD	8.8 ± 13.4	4.5 ± 5.3	0.33
Final indirect LLD (mm) Mean ± SD	9.4 ± 12.3	5.0 ± 4.6	0.28
Residual LLD >5 mm (n, %)	4 (31%)	7 (25%)	
Final MAD (mm) Mean ± SD	8.7 ± 5.9	7.2 ± 6.7	0.50
Residual MAD > 5 mm (n, %)	9 (69%)	12 (48%)	
Final mechanical axis angle angle^b^ Mean ± SD	3 ± 2	2.2 ± 2.1	0.26
Final LDFA (degrees) Mean ± SD	90.7 ± 5.7	89.1 ± 3.9	0.38
Final MPTA (degrees) Mean ± SD	88.9 ± 5.2	89.8 ± 2.8	0.58
BMAC to regenerate	2 (15%)	4 (14%)	
Yes (n, %)			

BHI = Bone Healing Index, BMAC = bone marrow aspirate concentrate, LDFA = lateral distal femoral angle, MAD = mechanical axis deviation, MAA = mechanical axis angle, MPTA = medial proximal tibial angle

^a^The two-sample Student *t*-test assuming unequal variances (two-tailed *P* values)

b Absolute value of MAD and MAA

Reported *P* values are two-tailed from the two-sample Student *t*-test assuming unequal variances

### Complications

One patient experienced one problem, and 13 patients experienced 17 obstacles (Table 4). There were no sequelae. The most common obstacle was delayed union of the regenerate, which was treated with percutaneous bone marrow aspirate concentrate (BMAC) injection as an outpatient procedure. The BMAC was obtained after percutaneous aspiration of 60cc from the iliac crest intramedullary space using the Harvest system (Terumo BCT). The three implant failures all occurred in one patient undergoing staged bilateral reconstructions. The first left Precice nail did not lengthen postoperatively and was exchanged. Then, the right one stopped lengthening, and the left Precice nail was not able to hold the ultimate length during consolidation. So, another surgery was done to replace the left nail to a trauma nail and the right one to a new Precice nail. Another patient fell 3 years after his treatment ended and sustained an ipsilateral femur shaft fracture. He underwent successful open reduction internal fixation, but this obstacle requiring another surgical intervention was counted for completeness. Given our numbers, no patient or treatment factors associated with obstacles were identified. All patients eventually achieved the preoperatively planned correction, and all Precice nails were electively removed after treatment end (typically one year after consolidation).

**Table 4:** Postoperative Problems and Obstacles Encountered After the Use of the Retrograde Magnetic Internal Lengthening Nail for Deformity Correction and Limb Lengthening

Problems	Patients (1)	Number (1)	Interventions
DVT	1	1	Medical therapy
**Obstacles**	**Patients (13)**	**Number (17)**	**Interventions**
Delayed union	5	6	BMAC injection
Nonunion	3	3	Exchange nailing
Implant failure^a^	1	3	Exchange nailing
Arthrofibrosis/soft-tissue contracture^b^	2	3	Open lysis of adhesions, gastrocnemius recession, and peroneal nerve decompression
Loss of alignment	1	1	Blocking screw insertion
Femur fracture after MILN removal	1	1	ORIF femur

BMAC = bone marrow aspirate concentrate, DVT = deep venous thrombosis, ORIF = open reduction and internal fixation, MILN = magnetic internal lengthening nails

a Includes left side twice and right side once in the same patient (see Results section)

b Includes right and left knees in one patient plus gastrocnemius recession and peroneal nerve decompression in another

## Discussion

Patient satisfaction with the treatment process is higher when limb lengthening reconstructions are accomplished with internal as opposed to external devices.^19^ Although there will always be a role for Ilizarov and hexapod frames for the correction of complex deformities, the fixator-assisted, blocking screw technique using a retrograde MILN facilitated acute deformity correction and gradual limb lengthening without needing postoperative external fixation. This study demonstrated the ability of this technique to correct substantial coronal plane malalignment, normalize the mechanical axis, and achieve all of the desired limb lengthening with an acceptable safety profile without complications that interfered with a patient's ultimate outcome.

The development of the LON technique shortened the time patients spent in an external fixator to achieve limb lengthening while improving bone consolidation times, improving physical therapy outcomes, and minimizing the risk of regenerate fracture during healing. The BHI for patients treated with femoral LON was 1.4 months/cm compared with a matched group treated with Ilizarov frames having 1.7 months/cm.^6^ Kocaoglu et al combined two techniques, LON and FAN, to correct femoral deformity and LLD. Deformity correction was done acutely and secured by the intramedullary nail, which was locked distally, and the same external fixator that was used for the deformity correction was then used for lengthening. They reported a BHI of 37 days/cm for an average of 6-cm lengthening. They were able to correct the mean MAD to 11 mm. Although these reported BHI and limb alignment corrections are similar to ours, the advantage of the retrograde MILN technique is in eliminating postoperative external fixation altogether.

LON remained the standard for femoral lengthening while earlier iterations of lengthening nails, such as the Intramedullary Skeletal Kinetic Distractor (Orthofix, Austin, TX) proved mechanically unreliable.^9,20^ A notable step forward for the field occurred with the development of the Precice nail, which became widely available after it proved to be reliable and accurate for bone lengthening.^21^ Our study further supports the accuracy and precision of the Precice nail for femoral lengthening. Nearly 100% of the preoperatively planned lengthening in our cohort was achieved, and measurements of the telescoping nail segment mirrored the cortical distraction gap. We urge caution in the interpretation of the final LLD measurements, which were based on radiographs after bone consolidation. Although radiographs suggested residual LLD >5 mm in four treated varus patients and seven valgus patients, slight soft-tissue contractures around the hip and knee that can develop during any lengthening treatment affect these 2-dimensional assessments. Therefore, we routinely stick to our preoperative plan to guide total lengthening with the expectation that measured leg lengths will equalize with postoperative physical therapy.

Although the Precice implant is more expensive than those used for LON, this cost is offset by the fewer surgical procedures that patients treated with the MILN typically undergo.^22^ Thus, it is becoming the standard implant for use in FAN procedures that address malalignment and LLD. Iobst et al^23^ reported their experience with retrograde MILN used to treat 27 patients with valgus, varus, and rotational malalignments plus LLD. This study demonstrated the ability to correct an average of 7° of angular deformity with a maximum of 15° corrected. Similar to our study, they were able to normalize the postoperative LDFA and MAD while achieving 100% of the desired limb lengthening. Their consolidation index was 42 days/cm, which is slightly longer than what we calculated for our cohort. Important technical findings from their article include that in the group of patients with a residual MAD ≥10 mm, smaller half pins (5 mm as opposed to 6 mm), fewer blocking screws (<2), and smaller diameter nails (8.5 mm or 10.7 mm versus 12.5 mm) were used. Although a stiffer fixator construct may better maintain acute deformity correction intraoperatively, we routinely loosen the fixator during nail insertion to allow the nail and blocking screws to work together to achieve ultimate deformity correction. If the intraoperative mechanical axis assessment is suboptimal, we would advocate for improving the correction by manipulating the half pins and creating a stiff fixator construct until the nail is interlocked.

The study by Iobst et al did not report any cases of delayed union despite having a cohort of similar age as ours and using similar postoperative nail distraction protocols^23^ while another similar study using the Fitbone internal lengthening nail (Wittenstein; Ingersheim, Germany) required bone grafting to the regenerate for delayed union in 9% of 22 cases.^24^ One challenge in comparing these findings directly with ours lies in the retrospective nature of each study where the definition for delayed union to guide surgical intervention was not formalized. For our series, an atrophic regenerate was treated with BMAC injection and classified as a delayed union even if it did not technically meet the FDA standard of absent radiographic progression of fracture healing over 3 months.^25^ Although the bone may have continued to full consolidation without intervention, a percutaneous BMAC injection carries minimal morbidity and is typically preferred in our patient population who desire an expeditious return to normal activities. Although the osteotomy gapping that occurs with these opening wedge corrections can theoretically inhibit regenerate formation, one study has shown that not to be the case.^26^ Our union rate may warrant additional study to see whether percutaneous bone grafting at the index procedure or a slower initial rate of nail distraction can decrease the need for subsequent surgical interventions. Another study has reported three nonunions requiring exchange nailing and bone grafting after five femur lengthenings with the Precice MILN, but this may be due to the fact that lengthening occurred in fewer increments (0.5 mm twice daily), and some cases involved the use of reamer-irrigator-aspirator systems, both of which make direct comparison with our study difficult.^27^

In addition to delayed union/nonunion, the second most common obstacle in our series was mechanical failure of the MILNs (3 instances in one patient). Previous studies have similarly noted mechanical failures of the Precice nail, which have included a failure of the lengthening mechanism to work postoperatively, unintentional loss of length during the consolidation phase, and fatigue fracture of the implant.^27-29^ Importantly, similar to this series, all of these mechanical complications were found in first-generation Precice nails. Since the second-generation Precice (P2) was released—which was designed with a solid housing—we have not experienced any mechanical failures.

## Limitations

Our study has several notable limitations. First, it is a retrospective study, thus limiting data gathering to that available in the EMR obtained during routine clinical care episodes. We did not define certain outcome measures, such as delayed union, beforehand. We also lack a control group for direct comparison of the retrograde MILN approach for the correction of limb malalignment and LLD, although multiple related series are available in the literature for adequate historical comparison.^6,12,23,24^ We also have a small sample size, thus making our data susceptible to the effect of possible outliers on the results. Finally, having patient-reported outcome measures before, during, and after treatment would strengthen this study and provide meaningful data to compare against that which is known about the patient experience with external fixators used for femoral deformity correction.

## Conclusions

The use of a retrograde MILN with a fixator-assisted, blocking screw technique is an effective means of acute deformity correction and gradual limb lengthening through minimal incisions. The accuracy of deformity correction hinges on intraoperative execution of the appropriate nail start site, osteotomy location, and placement of enough blocking screws to help stabilize the nail in the wide metadiaphysis of the distal femur.

## Supplementary Material

## References

[1] Tetsworth KD Paley D: Accuracy of correction of complex lower-extremity deformities by the Ilizarov method. Clin Orthop Relat Res 1994;301:102-110. 8156660

[2] Tsuchiya H Uehara K Abdel-Wanis ME Sakurakichi K Kabata T Tomita K: Deformity correction followed by lengthening with the Ilizarov method. Clin Orthop Relat Res 2002;402:176-183. 10.1097/00003086-200209000-00016 12218482

[3] Ilizarov GA: Clinical application of the tension-stress effect for limb lengthening. Clin Orthop Relat Res 1990;250:8-26. 2403497

[4] Ilizarov GA: The tension-stress effect on the genesis and growth of tissues: Part II. The influence of the rate and frequency of distraction. Clin Orthop Relat Res 1989;239:263-285. 2912628

[5] Liu Y Yushan M Liu Z Liu J Ma C Yusufu A: Complications of bone transport technique using the Ilizarov method in the lower extremity: A retrospective analysis of 282 consecutive cases over 10 years. BMC Musculoskelet Disord 2020;21:354. 32505174 10.1186/s12891-020-03335-w PMC7276072

[6] Paley D Herzenberg JE Paremain G Bhave A: Femoral lengthening over an intramedullary nail. A matched-case comparison with Ilizarov femoral lengthening. J Bone Joint Surg Am 1997;79:1464-1480. 9378732 10.2106/00004623-199710000-00003

[7] Rozbruch SR Kleinman D Fragomen AT Ilizarov S: Limb lengthening and then insertion of an intramedullary nail: A case-matched comparison. Clin Orthop Relat Res 2008;466:2923-2932. 18800209 10.1007/s11999-008-0509-8 PMC2628243

[8] Paley D: PRECICE intramedullary limb lengthening system. Expert Rev Med Devices 2015;12:231-249. 25692375 10.1586/17434440.2015.1005604

[9] Mahboubian S Seah M Fragomen AT Rozbruch SR: Femoral lengthening with lengthening over a nail has fewer complications than intramedullary skeletal kinetic distraction. Clin Orthop Relat Res 2012;470:1221-1231. 22143986 10.1007/s11999-011-2204-4 PMC3293955

[10] Fragomen AT Rozbruch SR: Retrograde magnetic internal lengthening nail for acute femoral deformity correction and limb lengthening. Expert Rev Med Devices 2017;14:811-820. 28893094 10.1080/17434440.2017.1378092

[11] Eidelman M Keren Y Norman D: Correction of distal femoral valgus deformities in adolescents and young adults using minimally invasive fixator-assisted locking plating (FALP). J Pediatr Orthop B 2012;21:558-562. 22960367 10.1097/BPB.0b013e328358f884

[12] Kocaoglu M Eralp L Bilen FE Balci HI: Fixator-assisted acute femoral deformity correction and consecutive lengthening over an intramedullary nail. J Bone Joint Surg Am 2009;91:152-159. 19122090 10.2106/JBJS.H.00114

[13] Fragomen AT Rozbruch SR: Lengthening of the femur with a remote-controlled magnetic intramedullary nail: Retrograde technique. JBJS Essent Surg Tech 2016;6:e20. 30237929 10.2106/JBJS.ST.15.00069 PMC6145623

[14] Paley D: Problems, obstacles, and complications of limb lengthening by the Ilizarov technique. Clin Orthop Relat Res 1990;250:81-104. 2403498

[15] Paley D Tetsworth K: Mechanical axis deviation of the lower limbs. Preoperative planning of uniapical angular deformities of the tibia or femur. Clin Orthop Relat Res 1992;280:48-64. 1611764

[16] Muthusamy S Rozbruch SR Fragomen AT: The use of blocking screws with internal lengthening nail and reverse rule of thumb for blocking screws in limb lengthening and deformity correction surgery. Strateg Trauma Limb Reconstr 2016;11:199-205. 10.1007/s11751-016-0265-3 PMC5069203 27665618

[17] Paley D Herzenberg JE Tetsworth K McKie J Bhave A: Deformity planning for frontal and sagittal plane corrective osteotomies. Orthop Clin North Am 1994;25:425-465. 8028886

[18] Moreland JR Bassett LW Hanker GJ: Radiographic analysis of the axial alignment of the lower extremity. J Bone Joint Surg Am 1987;69:745-749. 3597474

[19] Landge V Shabtai L Gesheff M Specht SC Herzenberg JE: Patient satisfaction after limb lengthening with internal and external devices. J Surg Orthop Adv 2015;24:174-179. 26688988

[20] Kenawey M Krettek C Liodakis E Wiebking U Hankemeier S: Leg lengthening using intramedullay skeletal kinetic distractor: Results of 57 consecutive applications. Injury 2011;42:150-155. 20638660 10.1016/j.injury.2010.06.016

[21] Kirane YM Fragomen AT Rozbruch SR: Precision of the PRECICE internal bone lengthening nail. Clin Orthop Relat Res 2014;472:3869-3878. 24682741 10.1007/s11999-014-3575-0 PMC4397804

[22] Richardson SS Schairer WW Fragomen AT Rozbruch SR: Cost comparison of femoral distraction osteogenesis with external lengthening over a nail versus internal magnetic lengthening nail. J Am Acad Orthop Surg 2019;27:e430-e436. 30278015 10.5435/JAAOS-D-17-00741

[23] Iobst CA Rozbruch SR Nelson S Fragomen A: Simultaneous acute femoral deformity correction and gradual limb lengthening using a retrograde femoral nail: Technique and clinical results. J Am Acad Orthop Surg 2018;26:241-250. 29494464 10.5435/JAAOS-D-16-00573

[24] Küçükkaya M Karakoyun Ö Sökücü S Soydan R: Femoral lengthening and deformity correction using the Fitbone motorized lengthening nail. J Orthop Sci 2015;20:149-154. 25326815 10.1007/s00776-014-0659-3 PMC4302230

[25] Thomas JD Kehoe JL: Bone Nonunion. Treasure Island (FL): StatPearls Publishing LLC., 2022. 32119272

[26] Karakoyun Ö Küçükkaya M Erol MF: Does lengthening after acute correction negatively affect bone healing during distraction osteogenesis? Acta Orthop Traumatol Turc 2015;49:405-409. 26312468 10.3944/AOTT.2015.14.0275

[27] Tiefenboeck TM Zak L Bukaty A Wozasek GE: Pitfalls in automatic limb lengthening - first results with an intramedullary lengthening device. Orthop Traumatol Surg Res 2016;102:851-855. 27527249 10.1016/j.otsr.2016.07.004

[28] Wiebking U Liodakis E Kenawey M Krettek C: Limb lengthening using the PRECICE(TM) nail system: Complications and results. Arch Trauma Res 2016;5:e36273. 28144605 10.5812/atr.36273 PMC5253187

[29] Schiedel FM Vogt B Tretow HL : How precise is the PRECICE compared to the ISKD in intramedullary limb lengthening? Reliability and safety in 26 procedures. Acta orthopaedica 2014;85:293-298. 24758320 10.3109/17453674.2014.913955 PMC4062798

