# Supplementary material for: Treatment of Angular Deformity and Limb Length Discrepancy With a Retrograde Femur Magnetic Intramedullary Nail: A Fixator-assisted, Blocking Screw Technique
Source: J Am Acad Orthop Surg Glob Res Rev. 2023 May 18;7(5):e23.00053. doi: 10.5435/JAAOSGlobal-D-23-00053 (PMC10566895; doi:10.5435/JAAOSGlobal-D-23-00053)
Supplement: Supplementary file 1 [file jagrr-7-e23.00053-s001.docx]

Supplemental Table 1: A comparison of preoperative to postoperative radiographic parameters within each study cohort.

|  | Varus Cohort (n=13) | |  |
| --- | --- | --- | --- |
|  | Preop | Postop | p-value |
| **Direct LLD (mm)** | 27.3 ± 18.8 | 8.8 ± 13.4 | **<0.001** |
| Mean +/- SD |  |  |  |
| **Indirect LLD (mm)** | 29.1 ± 15.6 | 9.4 ± 12.3 | **<0.001** |
| Mean +/- SD |  |  |  |
| **LLD > 5mm (n, %)** | 10 (77%) | 4 (31%) |  |
| **MAD (mm)** | medial (+) |  | **<0.001** |
| Mean +/- SD | 31.2 ± 21.5 | 8.7 ± 5.9 |  |
| **MAD > 5mm (n, %)** | 13 (100%) | 9 (69%) |  |
| **Mechanical axis angle** | deg. Varus (+) |  | 0.003 |
| Mean +/- SD | 10.4 ± 9.0 | 3 ± 2 |  |
| **LDFA (degrees)** | 98.4 ± 11.8 | 90.7 ± 5.7 | 0.001 |
| Mean +/- SD |  |  |  |
|  | | | |
|  | Valgus Cohort (n=28) | |  |
|  | Preop | Postop | p-value |
| **Direct LLD (mm)** | 25.2 ± 11.1 | 4.5 ± 5.3 | **<0.001** |
| Mean +/- SD |  |  |  |
| **Indirect LLD (mm)** | 26.3 ± 12.1 | 5 ± 4.6 | **<0.001** |
| Mean +/- SD |  |  |  |
| **LLD > 5mm (n, %)** | 25 (89%) | 7 (25%) |  |
| **MAD (mm)** | lateral (-) |  | **<0.001** |
| Mean +/- SD | -19 ± 8.7 | 7.2 ± 6.7 |  |
| **MAD > 5mm (n, %)** | 28 (100%) | 12 (42%) |  |
| **Mechanical axis angle** | deg. Valgus (-) |  | **<0.001** |
| Mean +/- SD | -6.2 ± 2.6 | 2.2 ± 2.1 |  |
| **LDFA (degrees)** | 82.5 ± 3.9 | 89.1 ± 3.9 | **<0.001** |
| Mean +/- SD |  |  |  |

P-values are reported as paired two-sample t-test for means (one-tailed P values)
